# Vitamin D Up-Regulates the Vitamin D Receptor by Protecting It from Proteasomal Degradation in Human CD4^+^ T Cells

**DOI:** 10.1371/journal.pone.0096695

**Published:** 2014-05-02

**Authors:** Martin Kongsbak, Marina R. von Essen, Lasse Boding, Trine B. Levring, Peter Schjerling, Jens P. H. Lauritsen, Anders Woetmann, Niels Ødum, Charlotte M. Bonefeld, Carsten Geisler

**Affiliations:** 1 Department of International Health, Immunology and Microbiology, Faculty of Health and Medical Sciences, University of Copenhagen, Copenhagen, Denmark; 2 Institute of Sports Medicine, Department of Orthopedic Surgery M, Bispebjerg Hospital and Center for Healthy Aging, Faculty of Health and Medical Sciences, University of Copenhagen, Copenhagen, Denmark; Medical University Vienna, Center for Brain Research, Austria

## Abstract

The active form of vitamin D_3_, 1,25(OH)_2_D_3_, has significant immunomodulatory properties and is an important determinant in the differentiation of CD4^+^ effector T cells. The biological actions of 1,25(OH)_2_D_3_ are mediated by the vitamin D receptor (VDR) and are believed to correlate with the VDR protein expression level in a given cell. The aim of this study was to determine if and how 1,25(OH)_2_D_3_ by itself regulates VDR expression in human CD4^+^ T cells. We found that activated CD4^+^ T cells have the capacity to convert the inactive 25(OH)D_3_ to the active 1,25(OH)_2_D_3_ that subsequently up-regulates VDR protein expression approximately 2-fold. 1,25(OH)_2_D_3_ does not increase VDR mRNA expression but increases the half-life of the VDR protein in activated CD4^+^ T cells. Furthermore, 1,25(OH)_2_D_3_ induces a significant intracellular redistribution of the VDR. We show that 1,25(OH)_2_D_3_ stabilizes the VDR by protecting it from proteasomal degradation. Finally, we demonstrate that proteasome inhibition leads to up-regulation of VDR protein expression and increases 1,25(OH)_2_D_3_-induced gene activation. In conclusion, our study shows that activated CD4^+^ T cells can produce 1,25(OH)_2_D_3_, and that 1,25(OH)_2_D_3_ induces a 2-fold up-regulation of the VDR protein expression in activated CD4^+^ T cells by protecting the VDR against proteasomal degradation.

## Introduction

In addition to its fundamental activity to maintain calcium and phosphorus homeostasis, the active form of vitamin D_3_, 1α,25-dihydroxyvitamin D3 (1,25(OH)_2_D_3_), has important immunomodulatory properties [1]. Epidemiological studies have shown that vitamin D deficiency is associated with higher risk of infections such as tuberculosis [2] and with increased risk of autoimmune diseases such as type 1 diabetes mellitus [3] and multiple sclerosis [4], [5]. Data from animal studies support a potential protective effect of vitamin D in autoimmune diseases [6]–[9], and the efficacy of high-dose vitamin D supplementation in patients with autoimmune diseases or infections is being tested in clinical trials [10], [11].

The biological actions of 1,25(OH)_2_D_3_ are mediated by the vitamin D receptor (VDR) that belongs to the nuclear hormone receptor superfamily [12], [13]. Interaction of 1,25(OH)_2_D_3_ with VDR induces heterodimerization with the retinoid X receptor (RXR) and translocation of 1,25(OH)_2_D_3_-VDR/RXR complexes into the nucleus [8], [14]–[17]. The 1,25(OH)_2_D_3_-VDR/RXR complexes bind to specific DNA sequences called vitamin D response elements (VDRE) in target genes, and dependent on the recruited co-regulators either augment or inhibit transcription of the target gene [17]–[19].

Responses to 1,25(OH)_2_D_3_ correlate with the VDR protein expression level in a given cell [20]–[22]. VDR expression varies with cell type and cellular differentiation, and is modulated by numerous stimuli including steroid and protein hormones, retinoids and growth factors such as epidermal growth factor, insulin and insulin-like growth factor [9], [23]. Furthermore, in some cell types VDR expression is modulated by the presence of its own ligand 1,25(OH)_2_D_3_. This type of receptor regulation has in some previous studies been called homologous regulation or auto-regulation. The typical response to 1,25(OH)_2_D_3_ is up-regulation of VDR expression. This can be caused by increased VDR gene transcription, concordant with the presence of VDRE in the VDR gene [24]–[29] and/or by stabilization of the VDR [22], [26], [30]–[35].

Naïve CD4^+^ T cells have the potential to differentiate into different types of effector cells that determine the nature of the immune response [36], [37]. One important determinant in the differentiation of CD4^+^ effector T cells is vitamin D. Thus, 1,25(OH)_2_D_3_ inhibits production of IFN-γ and augment the production of IL-4, thereby restraining Th1 differentiation and promoting Th2 differentiation, and furthermore, 1,25(OH)_2_D_3_ inhibits Th17 differentiation and induces differentiation of Treg [38]–[46]. Whether 1,25(OH)_2_D_3_ mediates its effect directly on CD4^+^ T cells or indirectly via APC or maybe by a combination of the two is still debated. If 1,25(OH)_2_D_3_ should have a direct effect of CD4^+^ T cells they must express the VDR. However, contradictory results have been reported concerning the expression of the VDR in human T cells. Most studies find that unstimulated T cells do not express the VDR, but that they start to express the VDR following activation with either lectins, antibodies against the T cell receptor (TCR), or phorbol esters in combination with ionomycin [47]–[56]. In contrast, some studies find that unstimulated T cells do express the VDR [57], [58]. These opposing results might be explained by the different subpopulations of leucocytes studied and the different methods for detection of the VDR applied. Only few studies have analyzed VDR expression in purified human CD4^+^ T cells and even here contradictory results have been reported. Thus, some studies find that unstimulated CD4^+^ T cells do not express the VDR but starts to express it following activation [49], [54], whereas other studies report that unstimulated CD4^+^ T cells do express the VDR [57].

Two studies have indicated that activation-induced VDR expression is augmented by 1,25(OH)_2_D_3_ in PBMC and T cells, respectively [52], [55]. In contrast, another study on purified CD4^+^ T cells found that unstimulated CD4^+^ T cells already express the VDR, and that neither activation nor 1,25(OH)_2_D_3_ induced up-regulation of the VDR, but that the combination did [57]. Thus, whether and how 1,25(OH)_2_D_3_ regulates VDR protein expression in CD4^+^ T cells remains to be determined.

As the VDR protein expression level is key for the cellular sensitivity to 1,25(OH)_2_D_3_, and 1,25(OH)_2_D_3_ influences the differentiation of CD4^+^ effector T cells, the aim of this study was to determine whether 1,25(OH)_2_D_3_ regulates VDR protein expression in human CD4^+^ T cells, and, if so, to elucidate the mechanisms behind this type of VDR regulation.

## Materials and Methods

### Chemicals and antibodies

25(OH)D_3_ (BML-DM-100-0001) and 1,25(OH)_2_D_3_ (BML-DM200-0050) were from Enzo Life Sciences, Inc., Ann Arbor, MI. Stock solutions of 2.5 mM 25(OH)D_3_ and 2.4 mM 1,25(OH)_2_D_3_ were prepared in anhydrous (≥99.5%) ethanol and stored at −80°C. To determine 1,25(OH)_2_D_3_ in the supernatants we used the 1,25-Dihydroxy Vitamin D EIA kit (AC-62F1) from IDS, Tyne and Wear, UK according to the manufacturer's instructions. Antibodies used included anti-VDR (D-6) and anti-CD3ζ (6B10.2) from Santa Cruz Biotechnology, Santa Cruz, CA, anti-p53 (9282) from Cell Signaling Technology, Danvers, MA, anti-GAPDH from (ab9485) from Abcam, Cambridge, MA and HRP-rabbit anti-mouse Ig (P0260) from DAKO, Glostrup, Denmark. Cycloheximide ready made solution 100 mg/ml in DMSO (C4859), phorbol 12-myristate 13-acetate (PMA, P8139), ionomycin (I0634), monensin (M5273), leptomycin B (LMB) (L2913) and ketoconazole (K1003) were from Sigma-Aldrich, St. Louis, MO. A fresh solution of ketoconazole 20 mg/ml in anhydrous ethanol was prepared before each experiment. The proteasome inhibitors lactacystin (426100) and MG-132 (474788) were from Merck Millipore, Nottingham, UK.

### Ethics statement, cell culture and T cell polarization

Mononuclear cells from blood were isolated by Lymphoprep (Axis-Shield, Oslo, Norway) density gradient centrifugation from healthy donors after obtaining informed, written consent in accordance with the Declarations of Helsinki principles for research involving human objects. The study was approved by The Committees of Biomedical Research Ethics for the Capital Region in Denmark (H-3-2009-132). Naïve CD4^+^ T cells were isolated using EasySep Human Naive CD4^+^ T cell Enrichment Kit (19155, Stemcell Technologies, Grenoble, France). The resulting cell population contained 95–98% CD4^+^ T cells of which more than 96% were CD45RA^+^. The purified naïve CD4^+^ T cells were cultured in serum-free X-VIVO 15 medium (1041, Lonza, Verviers, Belgium) at 37°C, 5% CO_2_ at a cell concentration of 1×10^6^ cells/ml in flat-bottomed 24-well tissue culture plates (142475) from Nunc, and stimulated with Dynabeads Human T-Activator CD3/CD28 beads (111.31D, Life Technologies, Grand Island, NY) at a cell to bead ratio of 5∶1 for 3 days. Cells present in the culture after 3 days were defined as activated T cells. In some experiments 25(OH)D_3_ or 1,25(OH)_2_D_3_ was added to the medium during the stimulation period. In polarization studies purified naïve CD4^+^ T cells were cultured and stimulated as described above in the presence of recombinant human IL-12 (5 ng/ml, 219-IL, R&D Systems) plus human IL-4 antibody (1 µg/ml, MAB204, R&D Systems) for Th1 polarization; in the presence of recombinant human IL-4 (10 ng/ml, 200–04, Peprotech) plus human IFN-γ antibody (1 µg/ml, MAB285, R&D Systems) for Th2 polarization and in recombinant human IL-1β (10 ng/ml, 201-LB, R&D Systems), recombinant human IL-6 (20 ng/ml, 206-IL, R&D Systems), recombinant human IL-23 (10 ng/ml, 1290-IL, R&D Systems) and recombinant human TGF-β1 (5 ng/ml, 240-B, R&D Systems) plus human IFN-γ antibody (1 µg/ml) and human IL-4 antibody (1 µg/ml) for Th17 polarization.

### Flow cytometry

After three days of stimulation the CD3/CD28 beads were removed from the cells, and the cells were re-stimulated with PMA (50 ng/ml) and ionomycin (500 ng/ml) in the presence of monensin (3 µM) as previously described [59]. The cells were then stained with PerCP/Cy5.5 anti-human CD4 (317428, BioLegend), fixed and permeabilized with BD Cytofix/Cytoperm followed by BD Perm/Wash according to the manufacturer instructions, and finally stained intracellularly with FITC mouse anti-human IFN-γ (554551, BD Pharmingen), anti-human IL-17A APC (17–7179, eBioscience), FITC Rat Anti-Human IL-4 (554484, BD Pharmingen) or PE anti-human IL-13 (501903, BioLegend). Data were acquired on a FACSCalibur (BD, Brøndby, Denmark) with CellQuest Pro software, and subsequently analyzed using FlowJo software.

### Western blot and regression analyses

For Western blot analysis, whole cell lysates were obtained by treatment of the cells with lysis buffer (50 mM Tris pH 7.5, 150 mM NaCl, 1 mM MgCl_2_) supplemented with 1% Triton X-100, 1× Protease inhibitor cocktail (P8340, Sigma-Aldrich) and 5 mM EDTA. The samples were run under reducing conditions on 10% polyacrylamide gels for 2 hours at 100 volt in 1× NuPAGE MOPS SDS Running buffer (XCell *SureLock* Mini-Cell Module, Life Technologies). For specific detection of proteins in the cytoplasmic and the nuclear fractions the NE-PER nuclear and cytoplasmic extraction reagents were used according to the manufacturer (78833, Thermo Fisher Scientific Inc., IL). An equal number of cells per lane were used for Western blot analysis regardless whether naïve or activated T cells were studied. The proteins were transferred to nitrocellulose membrane sheets (Amersham Bioscience) in 1× NuPAGE Transfer buffer supplemented with 10% methanol for 60 min at 40 volt (XCell II Blot Module, Life Technologies). The membranes were subsequently blocked for 60 min in Tris-buffered saline supplemented with 5% milk powder (Blotting Grade Blocker Non Fat Dry Milk, Bio-Rad) and 0.1% Tween 20 (P1379, Sigma-Aldrich) and incubated at 4°C for 24 hours with primary antibodies diluted in Tris-buffered saline supplemented with 5% bovine serum albumin (A4503, Sigma-Aldrich) and 0.1% Tween 20. The membranes were washed, and the proteins visualized following 60 min incubation at room temperature with secondary HRP-rabbit anti-mouse Ig using ECL (Amersham Biosciences) technology. The anti-VDR antibody recognized the VDR with an approximate m.w. of 50 kDa [54], anti-p53 recognized p53 with an approximate m.w. of 53 kDa, anti-GAPDH recognized GAPDH with an approximate m.w. of 40 kDa, and anti-CD3ζ recognized CD3ζ with an approximately m.w. of 16 kDa under reducing conditions [60]. For band density quantification ECL exposed sheets were analyzed in a ChemiDoc MP Imaging System from Bio-Rad. To determine the half-life, t½, of the VDR, cells activated in the absence or presence of 25(OH)D_3_ for 3 days were subsequently treated with the protein synthesis inhibitor cycloheximide for 0–4 hours, and the VDR protein expression levels determined by Western blot. The density of the bands were quantified and normalized to the density of the band of 25(OH)D_3_-treated cells at time zero. Exponential regression analysis on the mean relative band density from 3 independent experiments were performed by use of Microsoft Excel and defined as *D(t)  =  D(0) x e^−lt^*, where *D(t)* is the density at time *t*, *D(0)* is the initial density, i.e. the density at time t = 0, and *l* is the decay constant. The half-life was determined as *t½  =  ln(2)/l* and the mean VDR lifetime as *1/l*. To determine the increase in VDR protein expression following treatment of the cells with proteasome inhibitors, the bands were quantified and normalized to the density of the bands at time zero. Linear regression analysis on the mean relative band density from 3 independent experiments were performed by use of Microsoft Excel and defined as *D(t)  =  at + D(0)*, where *D(t)* is the density at time *t*, *D(0)* is the initial density, i.e. the density at time t = 0, and *a* is the coefficient of inclination.

### Real-time RT-PCR

mRNA for VDR and CYP24A1 were measured by real-time RT-PCR. For this, 2–5×10^6^ CD4^+^ T cells were lysed in TriReagent (Molecular Research Center) and 1-bromo-3-chloropropane (BCP) added to separate the sample into an aqueous and an organic phase. The RNA was precipitated from the aqueous phase using isopropanol, washed with ethanol and dissolved in RNase free water. Synthesis of complementary DNA (cDNA) was performed using 500 ng total RNA and Omniscript reverse transcriptase (Qiagen) in a total of 20 µl. cDNA was diluted 1∶10 in TE/salmon DNA buffer (10 mM Tris pH 8.0, 1 mM EDTA, 1 µg/ml Salmon testes DNA, D7656, Sigma-Aldrich), and 5 µl diluted cDNA (12.5 ng RNA) subsequently amplified (25 µl) in Quantitect SYBR Green Master Mix (Qiagen) with specific primers (100 nM) on a Stratagene MX3005P real-time PCR machine (Agilent Technologies). The thermal profile was set to 95°C for 10 min, followed by 50 cycles of amplification: 95°C for 15 s, 58°C for 30 s, 63°C for 90 s. Signal intensity was measured at the 63°C step and the threshold cycle (Ct) values were related to a standard curve made with known concentrations of DNA oligos (Ultramer oligos, Integrated DNA technologies, Leuven, Belgium) diluted in TE/salmon DNA buffer. After amplification reactions ran at 95°C for 60 s, 55°C for 30 s and heating slowly to 95°C to confirm specificity of the PCR products by melting curve analysis. Primers used for RT-PCR (sense/antisense primer) were:

VDR (CAGGCCCAACTCCAGACACACT/ATCCAGATTGGAGAAGCTGGACGA),

CYP24A1 (CCACGGGCAGAAGATTTGAGG/TTGTCAAGAGTCCGAGTTGTAAATGGT).

The data were normalized to number of cells by calculation from the total RNA yield per cell in each sample (the raw data represents number of target cDNA molecules measured per 12.5 ng total RNA).

### Statistical analysis

Statistical analyses were performed using Student's *t* test with a 5% significance level, paired observations and equal variance.

### Results

### Activated human CD4^+^ T cells produce 1,25(OH)_2_D_3_ and up-regulates VDR protein expression in the presence of 25(OH)D_3_

**Figure 1:**
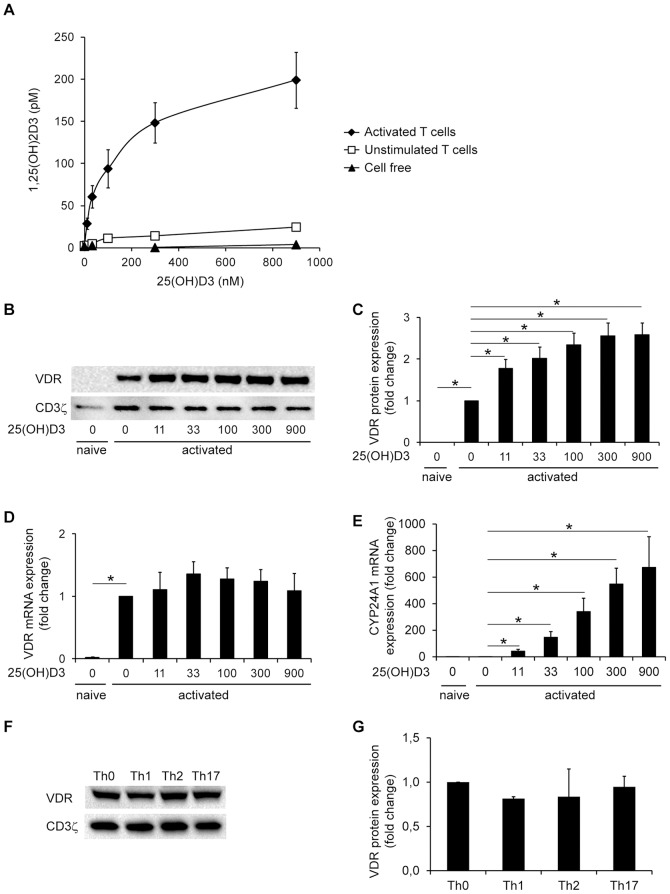
Activated human CD4^+^ T cells produce 1,25(OH)_2_D_3_ and up-regulates VDR expression in the presence of 25(OH)D_3_. (A) 1,25(OH)_2_D_3_ in the supernatants of activated and unstimulated T cells and in cell free cultures incubated with the indicated concentrations of 25(OH)D_3_. Mean ± SEM (*n* = 5). (B) Representative Western blot of VDR and CD3ζ (loading control) expression in naïve and activated T cells incubated in the presence of the indicated concentrations (nM) of 25(OH)D_3_. (C) Relative VDR protein expression as determined by the density of the VDR bands from Western blots of naïve and activated T cells incubated in the presence of the indicated concentrations (nM) of 25(OH)D_3_. The density of the VDR bands were normalized to the density of the VDR bands of T cells activated in the absence of 25(OH)D_3_. Mean + SEM (*n* = 7; * p<0.05). (D) Relative VDR mRNA expression in naïve and activated T cells incubated in the presence of the indicated concentrations (nM) of 25(OH)D_3_. The VDR mRNA levels were normalized to VDR mRNA levels of T cells activated in the absence of 25(OH)D_3_. Mean + SEM (*n* = 4; * p<0.001). (E) Relative CYP24A1 mRNA expression in naïve and activated T cells incubated in the presence of the indicated concentrations (nM) of 25(OH)D_3_. The CYP24A1 mRNA levels were normalized to CYP24A1 mRNA levels of T cells activated in the absence of 25(OH)D_3_. Mean + SEM (*n* = 3; * p<0.05). (F) Representative Western blot of VDR and CD3ζ (loading control) expression in T cells activated for 3 days in the presence of polarizing cytokines and anti-cytokine antibodies as indicated. (G) Relative VDR protein expression as determined by the density of the VDR bands from Western blots of T cells treated as described in F. The density of the VDR bands were normalized to the density of the VDR bands of T cells activated in the absence of polarizing cytokines and anti-cytokine antibodies (Th0). Mean + SEM (*n* = 2).

25-hydroxyvitamin D3 (25(OH)D_3_) is the inactive precursor of the active form of vitamin D3, 1,25(OH)_2_D_3_, and is considered the most reliant parameter when determining the vitamin D status of a subject. The normal range for serum concentrations of 25(OH)D_3_ is 25–170 nM, whereas the range for serum concentrations of 1,25(OH)_2_D_3_ is 60–110 pM, approximately 1000-fold lower than 25(OH)D_3_ [61]. It has been reported that T cells, especially following activation, express the 25(OH)D_3_ 1α-hydroxylase CYP27B1 that converts the inactive 25(OH)D_3_ to the active 1,25(OH)_2_D_3_; however, whether T cells can convert 25(OH)D_3_ to 1,25(OH)_2_D_3_ in physiological relevant concentrations is a matter of debate [55], [62]. To study whether 25(OH)D_3_ in physiological concentrations affects VDR expression, we first analyzed whether T cells actually had the ability to produce 1,25(OH)_2_D_3_ from 25(OH)D_3_ in our experimental setup. We purified naïve CD4^+^ T cells and either left them unstimulated or stimulated them with CD3/CD28 beads in the presence of increasing concentrations of 25(OH)D_3_. After 3 days we measured the concentration of 1,25(OH)_2_D_3_ in the supernatants. Activated T cells clearly had the ability to convert 25(OH)D_3_ to 1,25(OH)_2_D_3_ and produced significant amounts of 1,25(OH)_2_D_3_ compared to unstimulated T cells (Fig. 1A). In cell free control samples with 25(OH)D_3_ but without T cells, 1,25(OH)_2_D_3_ could not be detected (Fig. 1A). These results demonstrate that activated human CD4^+^ T cells have the capacity to produce 1,25(OH)_2_D_3_ from 25(OH)D_3_. To study how 1,25(OH)_2_D_3_ affects VDR expression levels, we determined VDR protein expression by Western blot analysis of T cells activated in the presence of increasing concentrations of 25(OH)D_3_. We found that T cell activation clearly induced VDR protein expression even in the absence of added 25(OH)D_3_ (Fig. 1B). Interestingly, 25(OH)D_3_ significantly increased the expression of the VDR in parallel with the 1,25(OH)_2_D_3_ production (Fig. 1A–C). Compared to T cells activated in the absence of 25(OH)D_3_, VDR protein expression was increased 2.0–2.3 fold in T cells activated in the presence of physiological concentrations of 25(OH)D_3_ at 33 – 100 nM (Fig. 1C). Naive T cells did not express the VDR, not even in the presence of 25(OH)D_3_ (Fig. 1B and data not shown).

These data demonstrated that T cell activation leads to VDR expression, and that presence of the VDR ligand further up-regulates VDR protein expression in activated T cells. To determine whether the 25(OH)D_3_-induced VDR up-regulation was caused by increased VDR gene transcription, we measured VDR mRNA expression by real-time RT-PCR in naïve T cells and in T cells activated in the absence or presence of 25(OH)D_3_. In accordance with the results obtained by the Western blot analyses, we found that naïve T cells express no or very low levels of mRNA for VDR, and that T cell activation strongly induced VDR gene transcription (Fig. 1D). However, addition of 25(OH)D_3_ did not significantly increase VDR mRNA expression in activated T cells (Fig. 1D). As control, we determined whether addition of 25(OH)D_3_ had any effect on classical 1,25(OH)_2_D_3_-responsive genes by measuring CYP24A1 mRNA in parallel with VDR mRNA. In contrast to the VDR mRNA, addition of 25(OH)D_3_ during T cell activation resulted in a massive up-regulation of CYP24A1 mRNA (Fig. 1D & E). From these experiments we could conclude that whereas 1,25(OH)_2_D_3_-responsive genes is strongly up-regulated in CD4^+^ T cells activated in the presence of 25(OH)D_3_, VDR gene transcription is not affected by the presence of 25(OH)D_3_ in CD4^+^ T cells.

Finally, to study whether polarization of activated CD4^+^ T cells towards the Th1, Th2 or Th17 lineage affected VDR expression we activated naïve CD4^+^ T cells with CD3/CD28 beads in the presence of IL-12 plus anti-IL-4 for Th1 polarization, IL-4 plus anti-IFN-γ for Th2 polarization and IL-1β, IL-6, IL-23 and TGF-β1 plus anti-IFN-γ and anti-IL-4 for Th17 polarization. As control, naïve T cells were activated in the absence of cytokines or anti-cytokines antibodies. In this experiment these control cells were termed Th0 cells. After 3 days of activation we determined VDR protein expression by Western blot analysis. We found that activated CD4^+^ T cells expressed the VDR at comparable levels independently of the polarization conditions. FACS analyses demonstrated that the cells were not fully polarized at this early time point, although cells polarized towards Th1 expressed more IFN-γ than cells polarized towards Th0, Th2 and Th17. Likewise cells polarized towards Th2 expressed less IFN-γ than cells polarized towards Th0 and Th1 (Figure **S1**). We could not detect IL-4 and IL-17 production at this time point.

Taken together, these experiments demonstrated that naïve CD4^+^ T cells neither express the VDR nor have the capacity to produce 1,25(OH)_2_D_3_ from 25(OH)D_3_. Shortly after activation with CD3/CD28 beads, they aquire the ability to produce 1,25(OH)_2_D_3_ from 25(OH)D_3_ and furthermore express the VDR independently of their polarization towards the Th0, Th1, Th2 or Th17 lineage.

### Ketoconazole inhibits 1,25(OH)_2_D_3_ production and 25(OH)D_3_-induced up-regulation of the VDR

**Figure 2:**
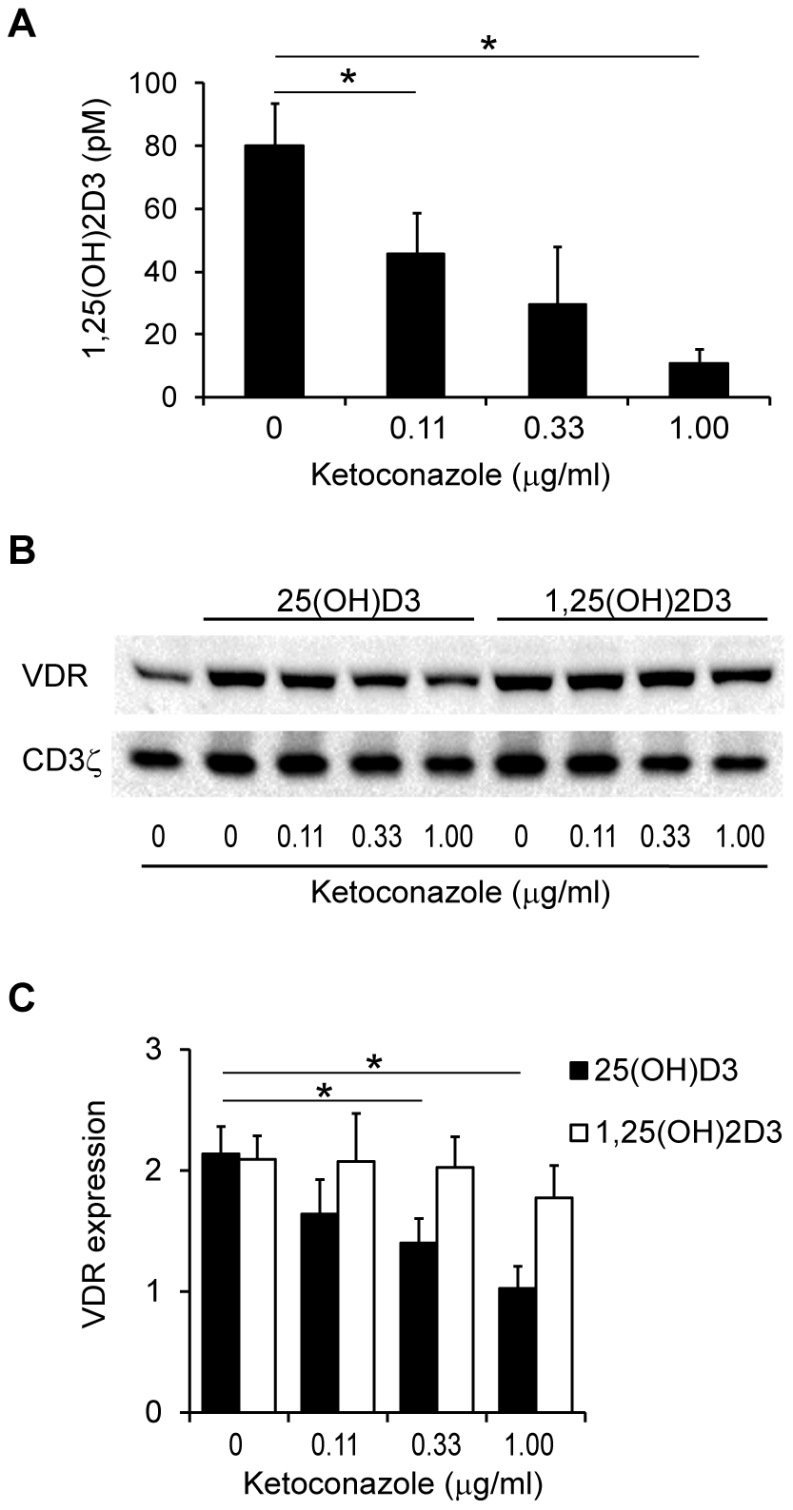
Ketoconazole inhibits 1,25(OH)_2_D_3_ production and up-regulation of the VDR. (A) 1,25(OH)_2_D_3_ in the supernatants of T cells activated in the presence of 100 nM of 25(OH)D_3_ and the indicated concentrations of ketoconazole. Mean + SEM (*n* = 3; * p<0.05). (B) Representative Western blot of VDR and CD3ζ (loading control) expression in T cells activated in the presence of the indicated concentrations of ketoconazole, 25(OH)D_3_ (100 nM) and 1,25(OH)_2_D_3_ (10 nM). (C) Relative VDR protein expression in T cells activated in the presence of the indicated concentrations of ketoconazole, 25(OH)D_3_ (100 nM) and 1,25(OH)_2_D_3_ (10 nM). The density of the VDR bands obtained by Western blot analysis were normalized to the density of the VDR bands of T cells stimulated in the absence of ketoconazole, 25(OH)D_3_ and 1,25(OH)_2_D_3_. Mean + SEM (*n* = 3; * p<0.01).

Although activated T cells in the presence of 25(OH)D_3_ clearly produced 1,25(OH)_2_D_3_, we could not exclude the possibility that the observed up-regulation of VDR was caused directly by 25(OH)D_3_ and not by 1,25(OH)_2_D_3_. To exclude this possibility, we examined the effect of the CYB27B1 antagonist ketoconazole [63] on 1,25(OH)_2_D_3_ production and up-regulation of the VDR. We activated T cells in the presence of 100 nM 25(OH)D_3_ and increasing concentrations of ketoconazole. After 3 days of incubation we measured the concentration of 1,25(OH)_2_D_3_ in the supernatants and the intracellular VDR protein expression levels. We found that ketoconazole efficiently inhibited the conversion of 25(OH)D_3_ to 1,25(OH)_2_D_3_ (Fig. 2A) and in parallel inhibited VDR up-regulation (Fig. 2B & C). These results indicated that ketoconazole inhibited 1,25(OH)_2_D_3_ production and thereby 1,25(OH)_2_D_3_-induced VDR up-regulation. If this was the case and ketoconazole did not inhibit VDR gene transcription, then addition of 1,25(OH)_2_D_3_ should rescue up-regulation of the VDR protein. Consequently, we activated T cells in the presence of 10 nM 1,25(OH)_2_D_3_ and increasing concentrations of ketoconazole. We found that in contrast to 25(OH)D_3_, exogenous 1,25(OH)_2_D_3_ induced up-regulation of the VDR protein in the presence of ketoconazole (Fig. 2B & C). These experiments indicated that it is 1,25(OH)_2_D_3_ and not 25(OH)D_3_ that increases VDR protein expression in activated T cells.

### 1,25(OH)_2_D_3_ increases the half-life of the VDR

**Figure 3:**
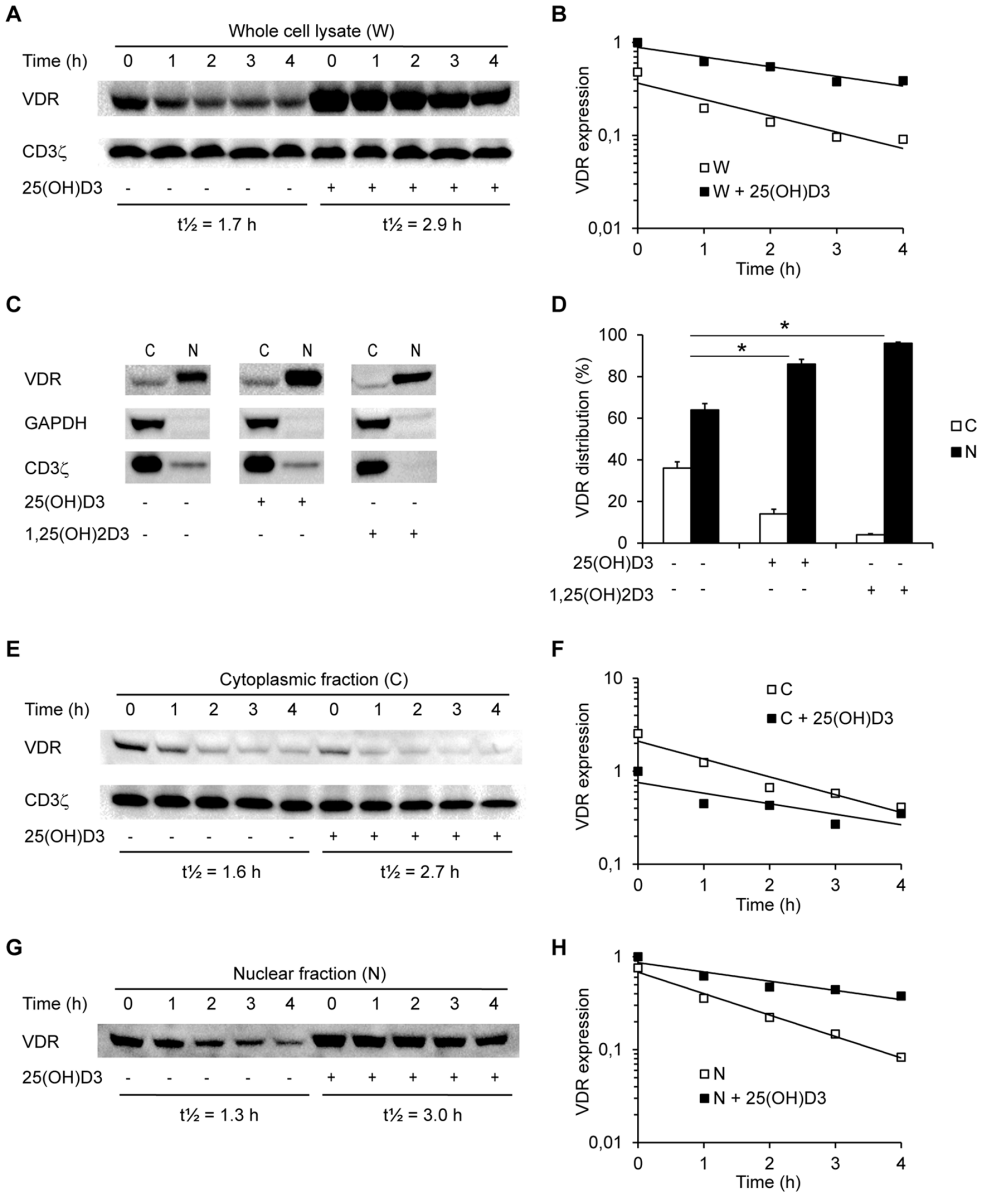
1,25(OH)_2_D_3_ increases the half-life of the VDR. (A) Representative Western blot of VDR and CD3ζ (loading control) in whole cell lysates from T cells activated in the absence or presence of 25(OH)D_3_ (100 nM) and then treated with cycloheximide (10 µg/ml) for the time indicated. The half-lives (t½) for the VDR in the absence or presence of 25(OH)D_3_ are given below the blot. (B) Relative VDR protein expression obtained from Western blot analysis of whole cell lysates from T cells activated in the absence (W) or presence (W +25(OH)D_3_) of 25(OH)D_3_ (100 nM) and then treated with cycloheximide (10 µg/ml) for the time indicated. The density of the VDR bands were normalized to the density of the VDR bands at time zero of T cells stimulated in the presence of 25(OH)D_3_. Shown are the mean relative densities from 3 independent experiments and the curves obtained by regression analysis for W (*D(t)  = 0.37e^−0.41t^*, R^2^  = 0.88) and W +25(OH)D_3_ (*D(t)  = 0.89e^−0.24t^*, R^2^  = 0.90). (C) Representative Western blot of VDR, GAPDH and CD3ζ (loading controls) expression in the cytoplasmic (C) and nuclear (N) fractions of T cells activated in the absence or presence of 25(OH)D_3_ (100 nM) and T cells activated in the absence of 25(OH)D_3_ and then treated for 4 h with 1,25(OH)_2_D_3_ (10 nM). (D) Distribution of the VDR in the cytoplasmic (C) and nuclear (N) fractions of T cells treated as described in (C), mean + SEM (n≥4; * p<0.01). (E) Representative Western blot of VDR and CD3ζ (loading control) expression in the cytoplasmic fraction of T cells treated as described in (A). (F) Relative VDR protein expression obtained from Western blot analysis of the cytoplasmic fraction from T cells activated in the absence (C) or presence (C +25(OH)D_3_) of 25(OH)D_3_ (100 nM) and then treated with cycloheximide (10 µg/ml) for the time indicated. The density of the VDR bands were normalized to the density of the VDR bands at time zero of T cells stimulated in the presence of 25(OH)D_3_. Shown are the mean relative densities from 3 independent experiments and the curves obtained by regression analysis for C (*D(t)  = 2.11e^−0.44t^*, R^2^  = 0.94) and C +25(OH)D_3_ (*D(t)  = 0.76e^−0.26t^*, R^2^  = 0.70). (G) Representative Western blot of VDR expression in the nuclear fraction of T cells treated as described in (A). (H) Relative VDR protein expression obtained from Western blot analysis of the nuclear fraction from T cells activated in the absence (N) or presence (N +25(OH)D_3_) of 25(OH)D_3_ (100 nM) and then treated with cycloheximide (10 µg/ml) for the time indicated. The density of the VDR bands were normalized to the density of the VDR bands at time zero of T cells stimulated in the presence of 25(OH)D_3_. Shown are the mean relative densities from 3 independent experiments and the curves obtained by regression analysis for N (*D(t)  = 0.68e^−0.53t^*, R^2^  = 0.99) and N +25(OH)D_3_ (*D(t)  = 0.86e^−0.23t^*, R^2^  = 0.90).

The above data indicated that 1,25(OH)_2_D_3_ mainly mediates VDR up-regulation by stabilization of the VDR. To directly determine how 1,25(OH)_2_D_3_ affects the half-life of the VDR, we activated T cells in the absence or presence of 100 nM 25(OH)D_3_ for 3 days. Subsequently, we treated the cells with the protein synthesis inhibitor cycloheximide for 0–4 hours and determined the VDR protein expression levels by Western blot analysis of whole cell lysates. We found that cycloheximide caused a gradual decrease in VDR protein expression with time in both untreated cells and cells treated with 25(OH)D_3_ (Fig. 3A). Regression analyses of the relative mean values of the density of the VDR bands gave the equations D(t)  = 0.37e^−0.41t^ with R^2^  = 0.88 and D(t)  = 0.89e^−0.24t^ with R^2^  = 0.90 for T cells activated in the absence and presence of 25(OH)D_3_, respectively (Fig. 3B). From these results, the half-life (t½) of the VDR was calculated to 1.7 h in untreated cells and 2.9 h in cells treated with 25(OH)D_3_ resulting in a mean VDR lifetime of 2.5 h and 4.2 h, respectively. Thus, we could conclude that 1,25(OH)_2_D_3_ up-regulates the VDR by increasing t½ and the mean lifetime of the VDR by approximately 1.7-fold.

Previous studies in other cell types than T cells have indicated that the VDR rapidly shuttles between the cytosol and the nucleus. The VDR is thus distributed to both the cytosol and the nucleus in the absence of 1,25(OH)_2_D_3_, and interaction of 1,25(OH)_2_D_3_ with the VDR shifts the localization of the VDR in favor of the nucleus in most but not all cell types studied [8], [15]–[17] To study the intracellular distribution of the VDR in T cells, we activated the cells in the absence or presence of 100 nM 25(OH)D_3_ for 3 days and subsequently determined the VDR protein expression levels in the cytoplasmic and nuclear fractions by Western blot analysis (Fig. 3C). We found that in the absence of 25(OH)D_3_ the VDR was distributed with approximately 35% in the cytoplasma and 65% in the nucleus, and that the presence of 25(OH)D_3_ induced a significant redistribution of the VDR resulting in localization of approximately 15% of the VDR in the cytoplasma and 85% in the nucleus (Fig. 3D). To investigate whether it actually was the active 1,25(OH)_2_D_3_ that caused the VDR distribution, we treated T cells that had been activated in the absence of 25(OH)D_3_ with 10 nM 1,25(OH)_2_D_3_ for the last 4 hours of the stimulation period and subsequently determined the VDR protein expression levels in the cytoplasmic and nuclear fractions. We found that approximately 95% of the VDR was located in the nucleus in T cells treated with 1,25(OH)_2_D_3_ (Fig. 3C and D), and we could conclude that 1,25(OH)_2_D_3_ induces a substantial redistribution of the VDR in activated T cells.

To study whether the 1,25(OH)_2_D_3_-induced redistribution of the VDR to the nucleus could explain the increased t½ of the VDR, we activated T cells in the absence or presence of 100 nM 25(OH)D_3_. Subsequently we treated them with cycloheximide for 0–4 hours and determined the VDR protein expression levels in the cytoplasmic and nuclear fractions separately by Western blot analysis. We found that the half-lives of the VDR were quite similar in the cytoplasma and nucleus, and that 1,25(OH)_2_D_3_ augmented the t½ of VDR to the same degree in both compartments (Fig. 3E–H). Thus, 1,25(OH)_2_D_3_ increased the t½ from 1.6 to 2.7 h in the cytosol and from 1.3 to 3.0 h in the nucleus.

### 1,25(OH)_2_D_3_ stabilizes the VDR by protecting it from proteasomal degradation

**Figure 4:**
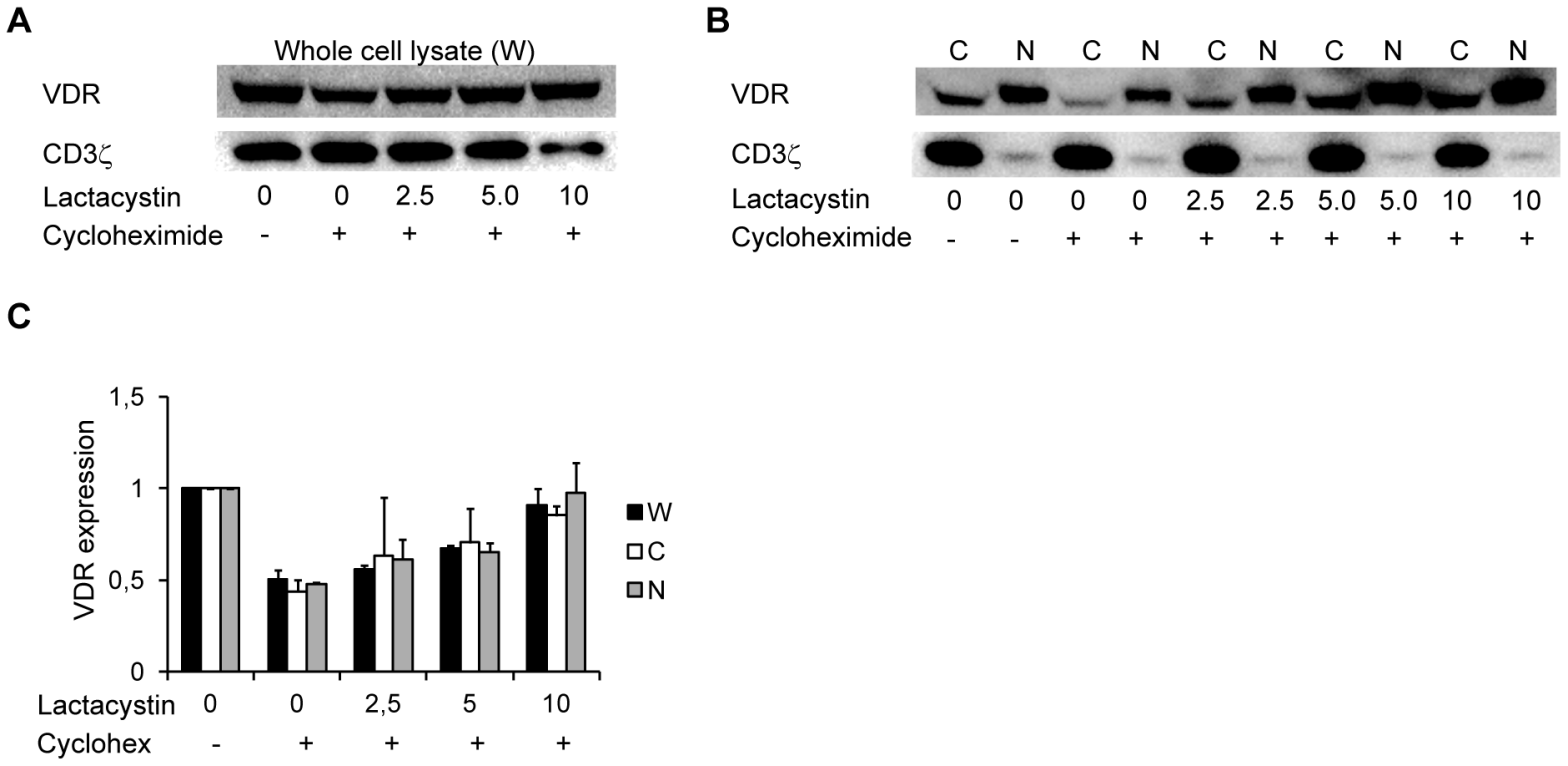
The VDR is spontaneously degraded by the proteasomes. (A, B) Representative Western blot of VDR and CD3ζ (loading control) in (A) whole cell lysates and (B) cytoplasmic (C) and nuclear (N) fractions of T cells activated for 3 d in the absence of 25(OH)D_3_ and then pre-treated with the indicated concentrations of lactacystin (µM) for 1 h before treatment with cycloheximide (10 µg/ml) as indicated for one additional h. (C) Relative VDR protein expression obtained from Western blot analysis of whole cell lysates (W), cytoplasmic (C) and nuclear (N) fractions from T cells treated as described in A and B. The density of the VDR bands were normalized to the density of the VDR bands of T cells not treated with lactacystin and cycloheximide. Results are presented as mean + SEM (*n* = 3).

To this point, our data indicated that the degradation rate of the VDR in human CD4^+^ T cells is regulated by 1,25(OH)_2_D_3_. Degradation of most cytosolic and nuclear proteins is carried out by the ubiquitin-proteasome pathway [64], [65]. To determine whether the VDR is degraded by the proteasomes in T cells, we activated the cells in absence of 25(OH)D_3_ for 3 days. Subsequently, we treated the cells with 0 to 10 µM of the proteasome inhibitor lactacystin for 1 hour, and then added cycloheximide for 1 additional hour. Finally, we determined the VDR protein expression levels by Western blot analysis of the whole cell lysates and the cytosolic and nuclear fractions (Fig. 4A & B). Cells treated with cycloheximide but without lactacystin expressed approximately 50% of the VDR compared to untreated cells in both whole cell lysates and the cytosolic and nuclear fractions (Fig. 4A–C) in agreement with a high VDR degradation rate in the absence of 1,25(OH)_2_D_3_. Increasing concentrations of lactacystin gradually rescued VDR protein expression, and inhibition of the proteasome with 10 µM lactacystin completely blocked VDR degradation (Fig. 4A–C). From these data we could conclude that in the absence of 1,25(OH)_2_D_3_ the VDR is spontaneously degraded by the proteasome.

**Figure 5:**
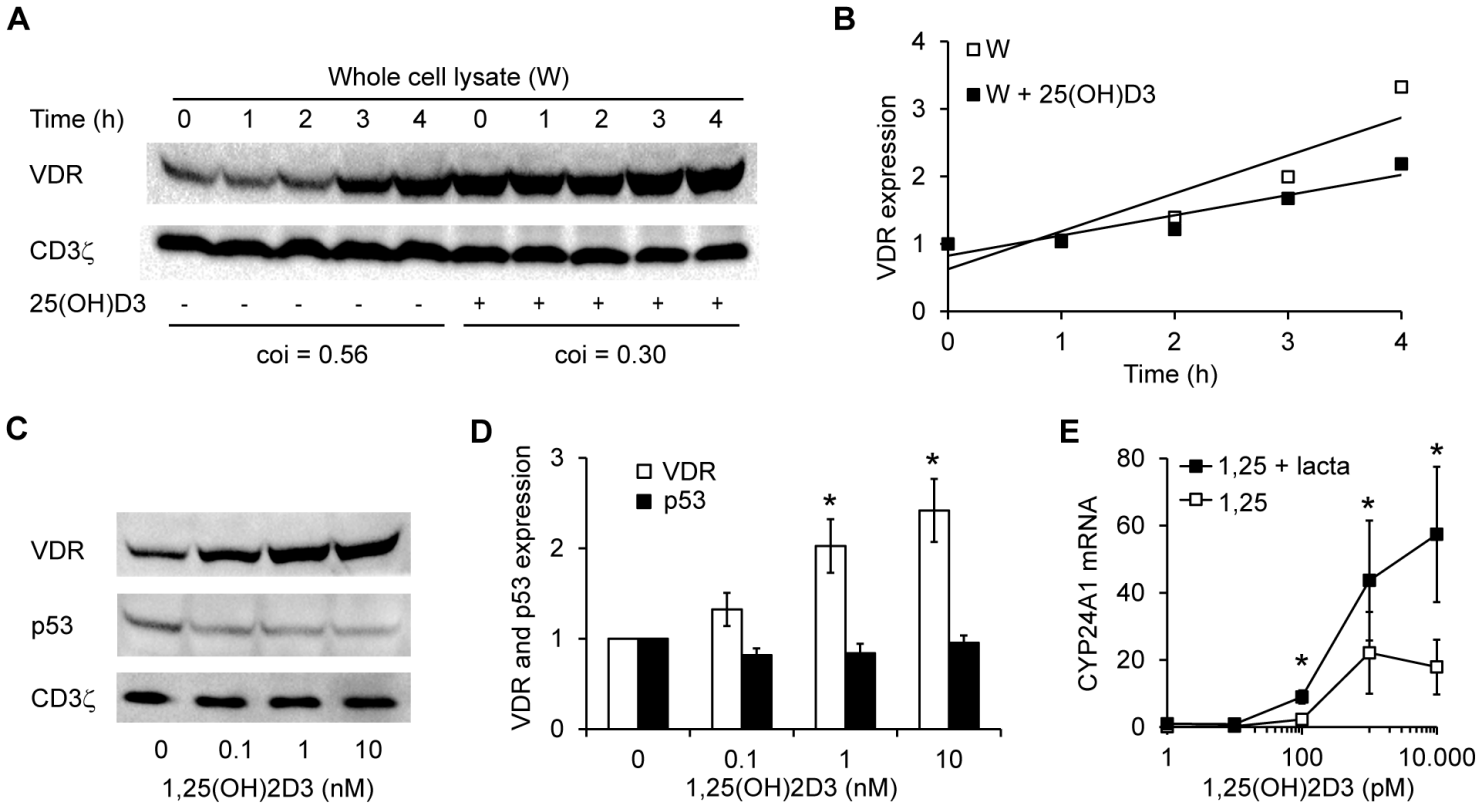
1,25(OH)_2_D_3_ stabilizes the VDR by protecting it from proteasomal degradation. (A) Representative Western blot of VDR and CD3ζ (loading control) expression in whole cell lysates of T cells activated for 3 d in the absence or presence of 25(OH)D_3_ (100 nM) and then treated with lactacystin (10 µM) for the time indicated. The coefficients of inclination (coi) obtained from the curves in B are given below the blots. (B) Relative VDR protein expression obtained from Western blot analysis of whole cell lysates (W) of T cells activated for 3 d in the absence or presence (+ 25(OH)D_3_) of 25(OH)D_3_ (100 nM). The density of the VDR bands were normalized to the density of the VDR bands at time zero of T cells activated in the absence or presence of 25(OH)D_3_, respectively. Shown are the mean relative densities from 3 independent experiments and the curves obtained by linear regression analysis of the mean values. (C) Representative Western blot of VDR, p53 and CD3ζ (loading control) in whole cell lysates of T cells activated for 3 d in the absence of 25(OH)D_3_ and then treated with the indicated concentrations of 1,25(OH)_2_D_3_ for 4 h. (D) Relative VDR and p53 protein expression obtained from Western blot analysis of whole cell lysates from T cells treated as described in C. The density of the VDR and p53 bands were normalized to the density of the VDR and p53 bands of T cells not treated with 1,25(OH)_2_D_3_, respectively. Results are presented as mean + SEM (*n* = 3; * p<0.05). (E) Relative CYP24A1 mRNA expression in T cells activated for 3 d in the absence of 25(OH)D_3_ and then treated with increasing concentrations of 1,25(OH)_2_D_3_ for 12 hours in the absence or presence of 10 µM lactacystin. The CYP24A1 mRNA levels were normalized to CYP24A1 mRNA levels of T cells not treated with1,25(OH)2D3. Results are presented as mean + SEM (*n* = 5; * p<0.05).

The above data suggested that 1,25(OH)_2_D_3_ induces VDR up-regulation by protecting the VDR against spontaneous degradation in the proteasome. If 1,25(OH)_2_D_3_ inhibits the proteasomal degradation of the VDR, it should be expected that the relative VDR protein expression levels increase more rapidly in the absence than in the presence of 1,25(OH)_2_D_3_ when proteasomal degradation is inhibited. To test this hypothesis, we activated T cells in the absence or presence of 100 nM 25(OH)D_3_. Subsequently, we treated the cells with lactacystin for 0–4 hours and determined the VDR protein expression levels (Fig. 5A). The density of the VDR bands were quantified and normalized to the density of the VDR bands at time 0. The relative VDR protein expression levels increased more rapidly in cells not treated with 25(OH)D_3_ than in cells treated with 25(OH)D_3_ (Fig. 5B). Similar results were obtained when the proteasome inhibitor MG-132 was used instead of lactacystin (data not shown). From these experiments we could conclude that 1,25(OH)_2_D_3_ protects the VDR against proteasomal degradation.

To investigate whether 1,25(OH)_2_D_3_ specifically protects the VDR against proteasomal degradation or whether 1,25(OH)_2_D_3_ inhibits proteasomal degradation in general, we simultaneously determined the expression levels of the VDR and the tumor suppressor protein p53, which normally is rapidly degraded by the proteasome [66]. We activated T cells in the absence of 25(OH)D_3_ for 3 days and subsequently treated the cells with increasing concentrations of 1,25(OH)_2_D_3_ for 4 hours. We then determined the levels of VDR and p53 by Western blot analysis of the whole cell lysates. As expected, we found that 1,25(OH)_2_D_3_ treatment resulted in increased levels of the VDR; however, 1,25(OH)_2_D_3_ did not affect p53 levels (Fig. 5C and D). Thus, we could conclude that 1,25(OH)_2_D_3_ specifically protects the VDR against proteasomal degradation.

To determine whether up-regulation of the VDR observed in cells treated with proteasome inhibitors had any physiological consequences for 1,25(OH)_2_D_3_-induced gene regulation, we activated T cells in the absence of 25(OH)D_3_ for 3 days. Subsequently, we treated the cells with increasing concentrations of 1,25(OH)_2_D_3_ for 12 hours in the absence or presence of the proteasome inhibitor lactacystin and then determined the CYP24A1 mRNA level. Cells treated with lactacystin were more sensitive to 1,25(OH)_2_D_3_ treatment. Thus, lactacystin-treated cells clearly started CYP24A1 gene transcription at lower concentrations of 1,25(OH)_2_D_3_ and showed significantly enhanced CYP24A1 transcription compared to cells not treated with lactacystin (Fig. 5E). Taken together, these experiments demonstrated that 1,25(OH)_2_D_3_ specifically protects the VDR against proteasomal degradation and that the response to 1,25(OH)_2_D_3_ correlates with the level of VDR protein expression in human CD4^+^ T cells.

### Leptomycin B neither inhibits nuclear export nor degradation of the VDR

**Figure 6:**
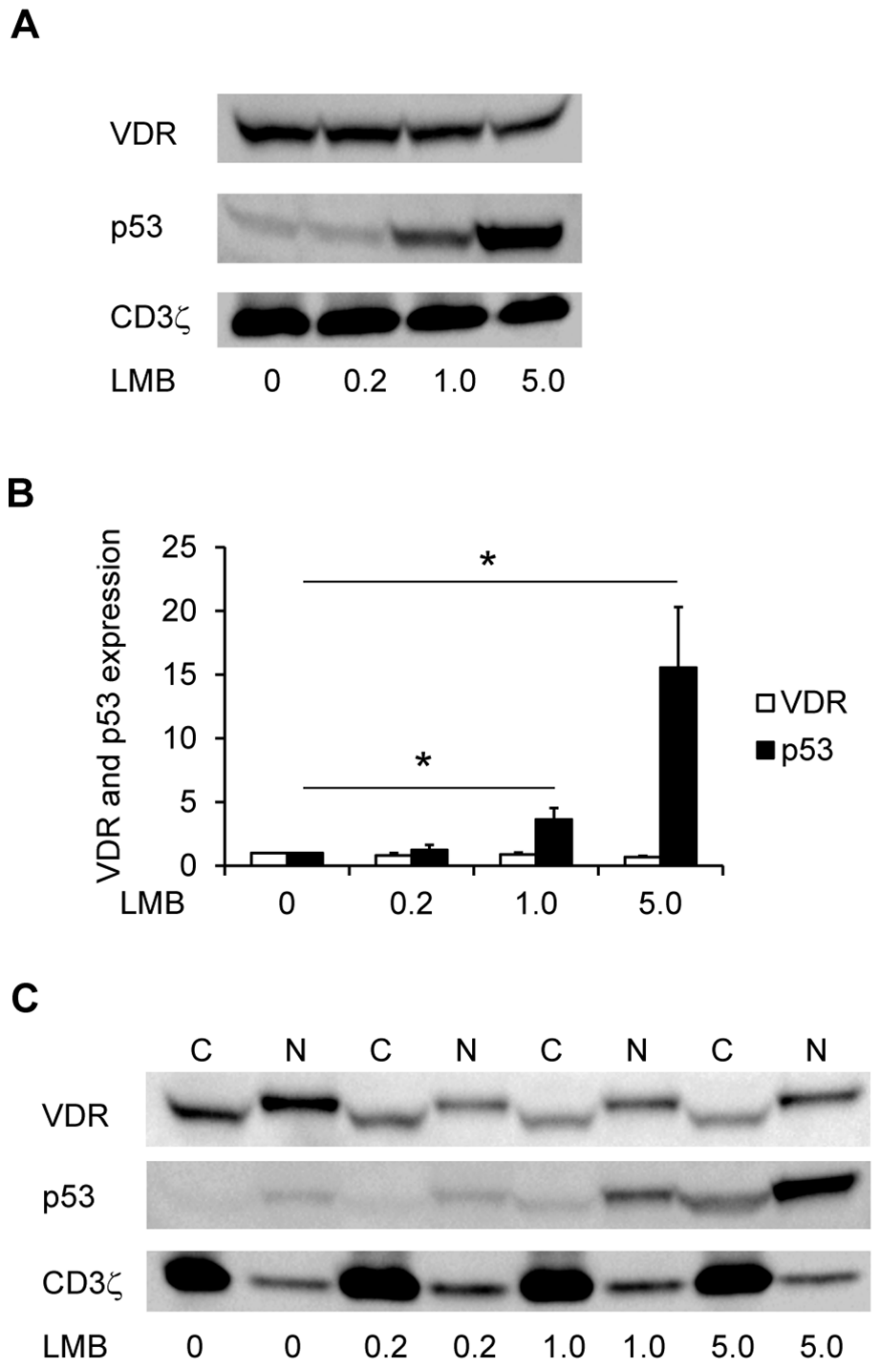
Leptomycin B neither inhibits nuclear export nor degradation of the VDR. (A) Representative Western blot of VDR, p53 and CD3ζ (loading control) in whole cell lysates of T cells activated for 3 d in the absence of 25(OH)D_3_ and then treated with the indicated concentrations (ng/ml) of leptomycin B (LMB) for 4 h. (B) Relative VDR and p53 protein expression obtained from Western blot analysis of whole cell lysates from T cells treated as described in A. The density of the VDR and p53 bands were normalized to the density of the VDR and p53 bands of T cells not treated with LMB, respectively. Results are presented as mean + SEM (*n* = 3; * p<0.05). (C) Representative Western blot of VDR, p53 and CD3ζ (loading control) in cytoplasmic (C) and nuclear (N) fractions of T cells treated as described in A.

From the results above it could be concluded that 1,25(OH)_2_D_3_ inhibits the proteasomal degradation of the VDR in human CD4^+^ T cells. At the same time 1,25(OH)_2_D_3_ induces translocation of the VDR from the cytosol to the nucleus. Previous studies in osteoblasts have suggested that the VDR is protected against proteasomal degradation in the nucleus [34], and this could also be the case for T cells. However, we found similar t½ for the VDR in the cytosol and the nucleus, and at first sight this indicated that translocation of the VDR to the nucleus did not explain the 1,25(OH)_2_D_3_-induced protection of the VDR. Yet, other studies have shown that the VDR rapidly shuttles between the cytosol and the nucleus [67], and at least two different scenarios could thus be envisioned: (i) 1,25(OH)_2_D_3_-induced protection of the VDR against proteasomal degradation is independent of VDR localization and takes place equally well in the cytosol and the nucleus, or (ii) the VDR is mainly degraded in the cytosol, and 1,25(OH)_2_D_3_ protects the VDR by affecting the cytoplasmic-nuclear shuttling in favor for localization of the VDR in the nucleus. To study which of these models that is valid in T cells, we set out to determine how blocking of the nuclear export of the VDR affected VDR stability. If scenario (i) was correct then blocking nuclear export should not affect the VDR protein expression level; however, if scenario (ii) was correct blocking nuclear export should lead to increased VDR levels. Leptomycin B (LMB) inhibits CRM1/exportin1 [68] and thereby blocks nuclear export of a variety of molecules including p53. p53 is normally exported from the nucleus to the cytoplasma where it is degraded, and treatment of cells with LMB consequently results in increased levels of p53 [66]. As it has been reported that LMB also blocks the export of unliganded VDR from the nucleus [67], we activated T cells in the absence of 25(OH)D_3_ for 3 days and subsequently treated the cells with increasing concentrations of LMB for 4 hours. We then determined the levels of VDR and p53 by Western blot analysis of the whole cell lysates. As expected, we found that LMB treatment resulted in increased levels of p53; however, LMB did not affect VDR levels (Fig. 6A and B). This suggested that scenario (i) was correct. To verify that LMB actually did block export of unliganded VDR from the nucleus, we determined the levels of VDR and p53 in the cytosolic and nuclear fractions of cells activated in the absence of 25(OH)D_3_ and subsequently treated with increasing concentrations of LMB. Surprisingly, unlike p53 the VDR did not accumulate in the nucleus after LMB treatment (Fig. 6C). From these results we could conclude that CRM1/exportin1 is not required for nuclear export of the VDR in T cells, and consequently we could not determine whether VDR in primary T cells is degraded in the cytosol, the nucleus or in both compartments.

## Discussion

In this study we determined the effect of 1,25(OH)_2_D_3_ on VDR expression in purified human CD4^+^ T cells activated with CD3/CD28 beads *in vitro*. We confirmed that naïve CD4^+^ T cells do not express the VDR. Activation of the CD4^+^ T cells induces VDR expression, and we found that 1,25(OH)_2_D_3_ further up-regulates the VDR protein expression approximately 2-fold by protecting the VDR against proteasomal degradation. Previous studies in other cell types have demonstrated that 1,25(OH)_2_D_3_ can up-regulate the VDR by increasing VDR mRNA expression [24]– and/or by stabilizing the VDR at the protein level [22], [26], [30]–[35]. Contradictory studies on VDR expression and the effect of 1,25(OH)_2_D_3_ on VDR expression in T cells have been published. Thus, two previous studies have indicated that activation-induced VDR expression is augmented by 1,25(OH)_2_D_3_ in PBMC and T cells, respectively [52], [55]. In contrast, another study found that unstimulated CD4^+^ T cells already express the VDR, and that neither activation nor 1,25(OH)_2_D_3_ induces up-regulation of the VDR, but that the combination does [57]. Thus, whether and how 1,25(OH)_2_D_3_ regulates VDR expression in CD4^+^ T cells has remained unknown until the present study.

To mimic physiological conditions, we incubate the cells with physiological concentrations of the precursor 25(OH)D_3_, which is found in 1000-fold higher concentrations in serum than 1,25(OH)_2_D_3_. We found that activated T cells can indeed convert 25(OH)D_3_ to the active 1,25(OH)_2_D_3_. The capacity of activated T cells to produce 1,25(OH)_2_D_3_ is in good agreement with studies demonstrating the expression of the 1α-hydroxylase CYP27B1 in activated T cells [55], [62]. In contrast to our results, Jeffery et al. found that human T cells did not have the capacity to produce 1,25(OH)_2_D_3_, although they found that T cell activation induced significant up-regulation of CYP27B1 [62]. We believe that this discrepancy can be explained by the fact that Jeffery et al. measured 1,25(OH)_2_D_3_ production after only 24 hours of T cell activation, whereas we measured it after 3 days of activation.

We found that 1,25(OH)_2_D_3_ up-regulates VDR protein expression approximately 2-fold in activated T cells without affecting VDR mRNA expression. As control we analyzed the effect of 25(OH)D_3_ on known 1,25(OH)_2_D_3_-responsive genes like CYP24A1 that became strongly up-regulated in CD4^+^ T cells activated in the presence of 25(OH)D_3_, while VDR gene transcription was unaffected by the presence of 25(OH)D_3_ in CD4^+^ T cells. This is in good agreement with observations in mouse fibroblasts and rat intestinal epithelial cells [30], the human breast cancer cell line T-47D [32], the human osteoblastic sarcoma cell line MG-63 [33], and the human keratinocyte cell line HaCaT [22], in which 1,25(OH)_2_D_3_ up-regulated VDR protein expression 2-3-fold without affecting VDR mRNA expression. Our results is also concordant with a previous study that found that 1,25(OH)_2_D_3_ up-regulated VDR expression in PBMC following activation; however, the types of cells that up-regulated the VDR was not identified in that study [52]. Our results are in contrast to the study by Baeke et al. which found that 1,25(OH)_2_D_3_ up-regulated VDR mRNA expression in activated T cells [55]. This discrepancy might be explained by the facts that Baeke et al. in contrast to us did not study purified subpopulations of T cells and furthermore used 1,25(OH)_2_D_3_ in concentrations more than 100 fold higher than physiological concentrations. Interestingly, a recent study found that 25(OH)D_3_ induced a 2-fold up-regulation in VDR mRNA expression in human monocytes [29]. Thus, the presence of monocyte in T cell preparations could confuse the results and might explain some of the inconsistent results on VDR regulation in T cells. Our results are also in contrast to a study by Veldman et al. which found that unstimulated CD4^+^ T cells already express the VDR, and that neither activation or 1,25(OH)_2_D_3_ induces up-regulation of the VDR, but that the combination does [57]. The discrepancy between our study and the study by Veldman et al. most probably can be explained by the different methods used to detect the VDR. Whereas we used the highly specific and sensitive anti-VDR antibody D-6 in Western blot analyses [69], Veldman et al. used a catching-ELISA with the IVG8C11 anti-VDR antibody produced against partially purified pig VDR [70] as the catching antibody. Later studies have demonstrated that IVG8C11 has extremely low sensitivity against the VDR [69], and thus the signals measured in the ELISA by Veldman et al. probably did not result from VDR binding.

By inhibiting CYP27B1 with ketoconazole we could block the conversion of 25(OH)D_3_ to 1,25(OH)_2_D_3_ and the up-regulation of the VDR protein expression in T cells activated in the presence of 25(OH)D_3_. In contrast, exogenous added 1,25(OH)_2_D_3_ still induced VDR protein up-regulation in the presence of ketoconazole. Although ketoconazole also inhibits other members of the cytochrome P450 superfamily, these results indicated that it is only the active form of vitamin D_3_ that has the potential to up-regulate the VDR. By blocking protein synthesis with cycloheximide we found that 1,25(OH)_2_D_3_ increases the half-life of the VDR in T cells by approximately 1.7-fold in accordance with previous studies in other cell types, which found that 1,25(OH)_2_D_3_ increased the VDR half-life approximately 2-fold [22], [30], [33].

We found that in the absence of 1,25(OH)_2_D_3_ the VDR distributes with approximately 35% in the cytosol and 65% in the nucleus in activated T cells. Addition of 1,25(OH)_2_D_3_ caused a significant redistribution of the VDR resulting in localization of more than 90% of the VDR in the nucleus. These findings extend prior studies in other cell types, which indicated that the VDR distributes evenly between the cytosol and the nucleus in the absence of 1,25(OH)_2_D_3_, and that 1,25(OH)_2_D_3_ facilitates translocation of the VDR to the nucleus [14]–[17]. It has been suggested that nuclear import of the VDR is important for stabilization of the VDR in osteoblasts [34]. The ubiquitin-proteasome pathway is the major route of disposal for most cytosolic and nuclear proteins [64], [65]. In agreement, our data demonstrated that human CD4^+^ T cells contain proteasome activity that degrades the VDR. Blocking proteasome activity increased the VDR levels to the same extent in the cytosol and nucleus. At first sight, this indicated that the VDR is degraded with similar kinetics in these compartments. However, the VDR most probably rapidly shuttles between the cytosol and the nucleus, and we could therefore not exclude that the VDR mainly is degraded in either the cytosol or the nucleus. To determine where the VDR is degraded, we studied the effect of LMB known to block nuclear export of a series of molecules [66], [68]. LMB has previously been reported to block nuclear export of unliganded VDR-GFP chimeras in transfected cell lines [67]; however, we clearly demonstrated that LMB neither inhibits nuclear export nor affects degradation of the VDR in CD4^+^ T cells. Consequently, we could not determine the primary site for VDR degradation, but we could conclude that 1,25(OH)_2_D_3_ inhibits the spontaneous proteasomal degradation of the VDR and thereby increases the half-life of the VDR in CD4^+^ T cells. These results are in good agreement with previous studies in other cell types, which found that 1,25(OH)_2_D_3_ inhibits ubiquitination and thereby proteasomal degradation of the VDR in the keratinocyte cell line HaCaT [22] and in Cos-1 cells [34]. 1,25(OH)_2_D_3_ might inhibit the proteasomal degradation of the VDR by inducing conformational changes of the VDR either directly or by promoting the association between VDR and RXR. Alternatively, 1,25(OH)_2_D_3_ might influence the expression of molecules involved in VDR degradation such as SUG1 [71] and CDK11B [72] and thereby affect VDR degradation. Future studies are required to precisely elucidate the mechanisms by which 1,25(OH)_2_D_3_ inhibits the proteasomal degradation of the VDR. Finally, we found that in parallel with up-regulation of VDR protein expression, proteasome inhibition leads to enhanced 1,25(OH)_2_D_3_-induced gene regulation. This is in good agreement with previous studies that found VDR up-regulation and enhanced sensitivity to 1,25(OH)_2_D_3_ following proteasome inhibition in keratinocytes and osteoblasts [22], [35].

Whereas most ligands desensitize their receptors, 1,25(OH)_2_D_3_ up-regulates its receptor and thereby increases the sensitivity of T cells for 1,25(OH)_2_D_3_. Combined with our observation that the VDR is expressed by all naïve T cells independently of the cytokine environment during the early stages of activation this substantiates that 1,25(OH)_2_D_3_ can play important roles in the early stages of T cell differentiation if found in sufficiently high local concentrations [38]–[46], [62].

In conclusion, our study establishes that naïve human CD4^+^ T cells do not express the VDR but that they start to express the VDR following stimulation via the TCR and CD28 independently of the presence of Th1, Th2 and Th17 polarizing cytokines. We further show that activated CD4^+^ T cells produce biological active concentrations of 1,25(OH)_2_D_3_ when provided with physiological concentrations of 25(OH)D_3_, and that 1,25(OH)_2_D_3_ induces a 2-fold up-regulation of VDR protein expression. We demonstrate that the 1,25(OH)_2_D_3_-induced VDR up-regulation is not caused by increased VDR mRNA expression but by protecting the VDR against proteasomal degradation. Finally we show that VDR up-regulation has functional consequences for 1,25(OH)_2_D_3_-responsive genes and thereby most probably consequences for CD4^+^ T cell differentiation and the ensuing immune response.

## Supporting Information

Figure S1 **IFN-γ expression in polarized CD4^+^ T cells activated for 3 days.** FACS plots of naïve CD4^+^ T cells activated for 3 days with CD3/CD28 beads in the presence of IL-12 plus anti-IL-4 for Th1 polarization, IL-4 plus anti-IFN-γ for Th2 polarization and IL-1β, IL-6, IL-23 and TGF-β1 plus anti-IFN-γ and anti-IL-4 for Th17 polarization. As control, naïve T cells were activated in the absence of cytokines or anti-cytokines antibodies (Th0 cells). The cells were stained for cell surface expression of CD4 and intracellular expression of IFN-γ and analyzed by flow cytometry.
(TIF)
