# Supplementary figures and images for: Vitamin D Up-Regulates the Vitamin D Receptor by Protecting It from Proteasomal Degradation in Human CD4+ T Cells
Source: PLoS One. 2014 May 2;9(5):e96695. doi: 10.1371/journal.pone.0096695 (PMC4008591; doi:10.1371/journal.pone.0096695)

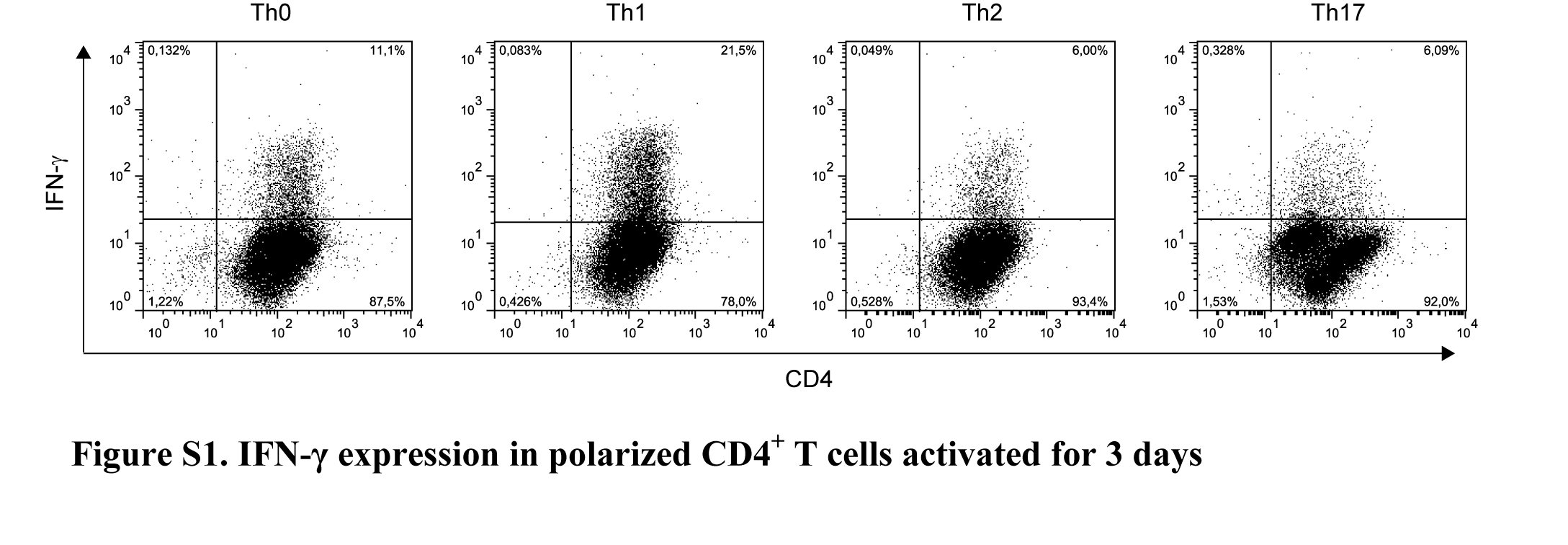

Supplement: Figure S1 — IFN-γ expression in polarized CD4+ T cells activated for 3 days. FACS plots of naïve CD4+ T cells activated for 3 days with CD3/CD28 beads in the presence of IL-12 plus anti-IL-4 for Th1 polarization, IL-4 plus anti-IFN-γ for Th2 polarization and IL-1β, IL-6, IL-23 and TGF-β1 plus anti-IFN-γ and anti-IL-4 for Th17 polarization. As control, naïve T cells were activated in the absence of cytokines or anti-cytokines antibodies (Th0 cells). The cells were stained for cell surface expression of CD4 and intracellular expression of IFN-γ and analyzed by flow cytometry. (TIF) [file pone.0096695.s001.tif]
